# Mutations in the Promoter and Coding Regions of *Avr3a* Cause Gain of Virulence of *Phytophthora sojae* to *Rps3a* in Soybean

**DOI:** 10.3389/fmicb.2021.759196

**Published:** 2021-11-11

**Authors:** Yanhong Hu, Zhihua He, Yebin Kang, Linkai Cui

**Affiliations:** College of Horticulture and Plant Protection, Henan University of Science and Technology, Luoyang, China

**Keywords:** Phytophthora root and stem rot, avirulence gene, escape mechanism, virulence variation, DNA mutations, molecular markers

## Abstract

*Phytophthora sojae* threatens soybean production worldwide, and the cultivation of soybean cultivars carrying *Rps* genes is the most effective way to control this pathogen. However, DNA mutations in the *Avr* genes of *P. sojae* can escape recognization of the corresponding *Rps* genes, leading to the loss of soybean resistance. In this study, we investigated sequence polymorphism and transcript level of the *Avr3a* gene in Chinese isolates of *P. sojae*. Twenty-four mutations resulting in five unique *Avr3a* alleles were discovered in the *Avr3a* coding region from 32 *P. sojae* isolates. The *Avr3a* transcripts were detectable in the isolates containing *Avr3a*(I), *Avr3a*(II), *Avr3a*(III), and *Avr3a*(IV) but not in the isolates containing *Avr3a*(V). Promoter and 5'-UTR sequence analysis revealed eight unique mutations in the promoter region of *Avr3a*(V), suggesting that the mutations could result in the loss of *Avr3a*(V) transcription. Virulence tests indicated the isolates containing *Avr3a*(II) and *Avr3a*(IV) were virulent, suggesting that the mutations in the coding regions of *Avr3a*(II) and *Avr3a*(IV) caused the gain of virulence to *Rps3a*. Based on DNA mutations of *Avr3a* in virulent alleles, two SNP markers and one PCR-based marker were developed successfully for detecting the virulence of *P. sojae* isolates to *Rps3a*. These findings provide new insights into escape mechanisms of *Avr3a* and effective support for accurate pathotype identification of *P. sojae* using molecular methods.

## Introduction

*Phytophthora sojae* Kaufmann & Gerdemann is an oomycete of the Stramenopila Kingdom and a soilborne pathogen of soybean. The pathogen causes damping-off of soybean seedlings and root and stem rot of adult plants, known as Phytophthora root and stem rot (PRR) of soybean. PRR is a devastating disease of soybean worldwide and causes approximately $1–2 billion in annual agricultural losses (Tyler, 2007). Host resistance, soil drainage control, and fungicidal seed treatments, such as metalaxyl, are generally used to control the disease (Dorrance et al., 2009). The most effective way to control PRR currently involves growing soybean cultivars carrying resistance genes to *P. sojae* (*Rps* genes) (Sahoo et al., 2017; Sukumaran et al., 2018; Zhong et al., 2019).

The interaction between *P. sojae* and soybean follows the gene-for-gene model. The avirulence (*Avr*) genes of *P. sojae* determine the efficacy of the *Rps* genes in soybean. Nine *Avr* genes have been cloned from *P. sojae* to date: *Avr1b*-1 (Shan et al., 2004), *Avr1a* and *Avr3a/5* (Qutob et al., 2009), *Avr3c* (Dong et al., 2009), *Avr4/6* (Dou et al., 2010), *Avr3b* (Dong et al., 2011a), *Avr1k* (Song et al., 2013), *Avr1d* (Yin et al., 2013), and *Avr1c* (Na et al., 2014). These *Avr* genes encode secreted proteins with RXLR-dEER amino acid motifs. Avr proteins can be recognized by the corresponding Rps proteins in soybean, resulting in effector-triggered immunity. However, under the selective pressure of *Rps* genes, *P. sojae* can escape recognition by soybean through DNA modifications such as substitutions, frameshift mutations, large insertions, deletions, recombinations, or changes in expression of the *Avr* genes (Tyler and Gijzen, 2014; Zhang et al., 2019).

The large-scale deployment of *Rps* genes in soybean has led to the rapid evolution of the virulence profile (pathotype) of *P. sojae* populations, accompanying by the ineffectiveness of the deployed *Rps* genes (Tremblay et al., 2021). In order to determine and deploy the proper *Rps* genes, it is very important that the pathotypes of *P. sojae* are identified accurately. At present, the pathotypes of *P. sojae* are determined mainly through virulence tests using a soybean differential set (Cui et al., 2010; Guérin et al., 2014; Lebreton et al., 2018). As the bioassay is time-consuming and labor-intensive, it is not quite suitable for a large-scale virulence test. Hence, the development of simple and rapid molecular diagnostic methods is the main tendency for pathotype identification. More recently, Dussault-Benoit et al. (2020) developed a molecular assay that could define *P. sojae* pathotypes using seven *Avr* genes (*Avr1a*, *Avr1b*, *Avr1c*, *Avr1d*, *Avr1k*, *Avr3a*, and *Avr6*). The matching rate between the molecular assay and the phenotyping assay was as high as 97% (170/175). This molecular assay offers a powerful tool for the selection of germplasm resistant to *P. sojae*.

Although the molecular assay has been developed successfully, it is not very perfect. Five cases of discrepancy between the molecular assay and the phenotyping assay were observed in their study. Interestingly, all five cases of discrepancy were present in the *Avr3a* gene. The PCR amplification for the avirulent alleles of *Avr3a* was achieved in the five cases, but the compatible interactions with *Rps3a* were observed in the phenotyping assay (Dussault-Benoit et al., 2020). *Avr3a* is a RXLR effector gene and encodes a predicted protein of 111 amino acids that includes a signal peptide, a RXLR motif and a carboxyterminal effector domain (Qutob et al., 2009; Dong et al., 2011b). Rps3a in soybean can recognize Avr3a to generate an incompatible reaction. So far, only three alleles of *Avr3a* have been identified in *P. sojae* field populations, based on which the PCR assay was developed. The cases of discrepancy suggest that there may be unknown virulent alleles of *Avr3a* in natural populations that can be amplified by the PCR assay.

Therefore, it is necessary for us to do further investigations and improve the molecular assay for the perfect match between the molecular assay and the phenotyping assay. In this study, we analyzed the polymorphisms in the promoter and coding regions of *Avr3a* in Chinese *P. sojae* isolates and measured the transcript levels of each allele of *Avr3a* in order to: (i) discover novel alleles of *Avr3a*, (ii) analyze how *Avr3a* escape recognition by *Rps3a* in China, and (iii) develop novel and reliable molecular markers for accurate differentiation of virulent alleles from avirulent alleles.

## Materials and Methods

### *Phytophthora sojae* Isolates and Growth Conditions

A total of 32 *P. sojae* isolates were used in this study and purified by single zoospore isolation (Table 1). All isolates were recovered from soybean field soils in China (Cui et al., 2010, 2012). These isolates were grown on 10% V8 juice agar at 25°C in the dark for virulence tests and cultured in 10% V8 juice at 25°C in the dark for mycelium collection.

**Table 1:** Virulence of *Phytophthora sojae* isolates to *Rps3a* and the *Avr3a* alleles.

Isolate	Origin			Virulence test^1^		Type of *Avr3a*^2^
	Source	Sampling location	Year	L83-570 (*Rps3a*)	Willams (*rps*)	
Ps0402	Soil	Heilongjiang, China	2004	A	V	I
Ps0702	Soil	Sicuan, China	2007	A	V	I
Ps0704	Soil	Jiangsu, China	2007	A	V	I
Ps0707	Soil	Heilongjiang, China	2007	A	V	I
Ps0708	Soil	Xinjiang, China	2007	A	V	I
Ps0712	Soil	Guizhou, China	2007	A	V	I
Ps0301	Soil	Heilongjiang, China	2003	V	V	II
Ps0714	Soil	Henan, China	2007	V	V	II
Ps0705	Soil	Fujian, China	2007	A	V	III
Ps0710	Soil	Fujian, China	2007	A	V	III
Ps0901	Soil	Fujian, China	2007	A	V	III
Ps0902	Soil	Heilongjiang, China	2007	A	V	III
Ps0905	Soil	Jilin, China	2009	A	V	III
Ps0906	Soil	Jilin, China	2009	A	V	III
Ps0907	Soil	Henan, China	2009	A	V	III
Ps0903	Soil	Helongjiang, China	2007	V	V	IV
Ps0904	Soil	Henan, China	2007	V	V	IV
Ps0302	Soil	Heilongjiang, China	2003	V	V	V
Ps0303	Soil	Heilongjiang, China	2003	V	V	V
Ps0401	Soil	Heilongjiang, China	2004	V	V	V
Ps0404	Soil	Heilongjiang, China	2004	V	V	V
Ps0405	Soil	Heilongjiang, China	2004	V	V	V
Ps0406	Soil	Heilongjiang, China	2004	V	V	V
Ps0701	Soil	Henan, China	2007	V	V	V
Ps0709	Soil	Henan, China	2007	V	V	V
Ps0716	Soil	Henan, China	2007	V	V	V
Ps0719	Soil	Sichuan, China	2007	V	V	V
Ps0720	Soil	Sichuan, China	2007	V	V	V
Ps0908	Soil	Anhui, China	2009	V	V	V
Ps0909	Soil	Anhui, China	2009	V	V	V
Ps0910	Soil	Jiangsu, China	2009	V	V	V
Ps0911	Soil	Jiangsu, China	2009	V	V	V

1 *A isolate was considered to be virulent (V) if≥70% of soybean seedlings were killed and avirulent (A) if≤30% of soybean seedlings were killed*.

2 *Five Avr3a alleles were identified and designated as Avr3a(I), Avr3a(II), Avr3a(III), Avr3a(IV), and Avr3a(V)*.

### Virulence Tests

Soybean cultivars Williams (*rps*) and L83-570 (*Rps3a*) were used to evaluate the virulence of *P. sojae* isolates by hypocotyl split inoculation, as described previously (Cui et al., 2010). *Phytophthora sojae* isolates were grown on V8 plates for a week. Each treatment contained at least 10 plants, and each virulence test was performed in triplicate. An isolate was considered to be virulent if ≥70% of soybean seedlings were killed, avirulent if ≤30% of soybean seedlings were killed, and intermediate if 30–70% of soybean seedlings were killed (Ryley et al., 1998).

### DNA Extraction, PCR Amplification, Cloning, and Sequence Analysis

Genomic DNA was isolated from mycelial cultures of *P. sojae* isolates following the protocol described by Tyler et al. (1995). PCR reactions were conducted in a total volume of 50μl containing 50ng template DNA, 2U PrimeSTAR HS DNA Polymerase (Takara, Dalian, China), 200μM dNTPs, 300mM of each primer, and 1×PrimeSTAR Buffer (Mg^2+^ Plus). The sequences of primers are shown in Supplementary Table S1. PCR was performed in a TP600 Thermal Cycler (Takara) under the following PCR program: 1cycle at 98°C for 1min for initial denaturation, 30cycles at 98°C for 10s, 56°C for 15s, 72°C for 1min, and a final 5min extension at 72°C. The PCR products were purified using a MiniBEST Agarose Gel DNA Extraction Kit (Takara), cloned into pMD19-T vectors (Takara), and sequenced by Sangon Biotech Co., Ltd. in Shanghai, China. Three clones were sequenced for each isolate. Sequence analysis was conducted using Bioedit 7.0 and Mega 7.0 softwares.

### RNA Extraction and Transcript Level Analysis

Total RNA was extracted from *P. sojae*-infected soybean leaves as described by Ye et al. (2011). Total RNA of leaves at 12h post-inoculation was isolated and treated with RNase-free DNase I (Takara) to remove genomic DNA. The integrity of total RNA was assessed by agarose gel electrophoresis. Single-stranded cDNA was synthesized using MMLV reverse transcriptase (Takara) and oligo (dT)18 primer. Real-time reverse transcription (RT)-PCR was conducted in 20μl reactions including 20ng of cDNA, 0.2μM of a gene-specific primer for *Avr3a* or reference *Actin* gene (Supplementary Table S1), 10μl 2×TB Green Premix Ex Taq (Takara) to measure the transcript levels of five alleles of *Avr3a* in *P. sojae* isolates from China. Reactions were carried out on an ABI 7300 real-time PCR system (Applied Bio-systems, Foster City, CA, United States) under the following conditions: 95°C for 30s, 40cycles of 95°C for 5s, and 60°C for 31s to calculate cycle threshold (Ct) values, followed by a dissociation program of 95°C for 15s, 60°C for 1min, and 95°C for 15s to obtain melt curves. The relative expression level of each sample was determined using 7,300 system sequence detection software. The real-time RT-PCR assay was repeated three times, each with three independent biological replicates.

### PCR Detection of Virulence

A PCR assay was conducted to detect the virulence of *P. sojae* to *Rps3a* as described by Dussault-Benoit et al. (2020). Briefly, the PCR was performed in a total volume of 25μl including 1×Taq Master Mix (Nobelab, Beijing, China), 400mM of forward and reverse primer, and 50ng template DNA. PCR amplifications were carried out in a TP600 Thermal Cycler with the following conditions: 1cycle at 94°C for 2min for initial denaturation, 32cycles at 94°C for 30s, 62°C for 30s, 72°C for 30s, and 2min of final extension at 72°C. PCR products were separated on 1.5% agarose gels in 0.5×TBE buffer, stained with 4S Green Nucleic Acid Stain (Sangon Biotech, Shanghai, China), and visualized using a Gel Doc XR+UV transilluminator (Bio-Rad, Hercules, CA, United States). The presence of an amplification product at the expected size was regarded as an avirulent isolate and vice versa.

## Results

### The Virulence of *Phytophthora sojae* Isolates From China on the *Rps3a* Soybean

The virulence of 32 *P. sojae* isolates from China was evaluated by hypocotyl split inoculation. All isolates were virulent on the soybean cultivar Williams (*rps*). Thirteen isolates were avirulent on the soybean cultivar L83-570 (*Rps3a*) and the other 19 were virulent (Table 1). Of the isolates, 59.38% were virulent on the *Rps3a* soybean, suggesting that most isolates are able to escape recognization of *Rps3a* in China and the *Rps3a* gene is not an effective *Rps* gene for controlling Phytophthora root and stem rot.

### Polymorphism Analysis of *Avr3a* in *Phytophthora sojae* Isolates From China

**Figure 1:**
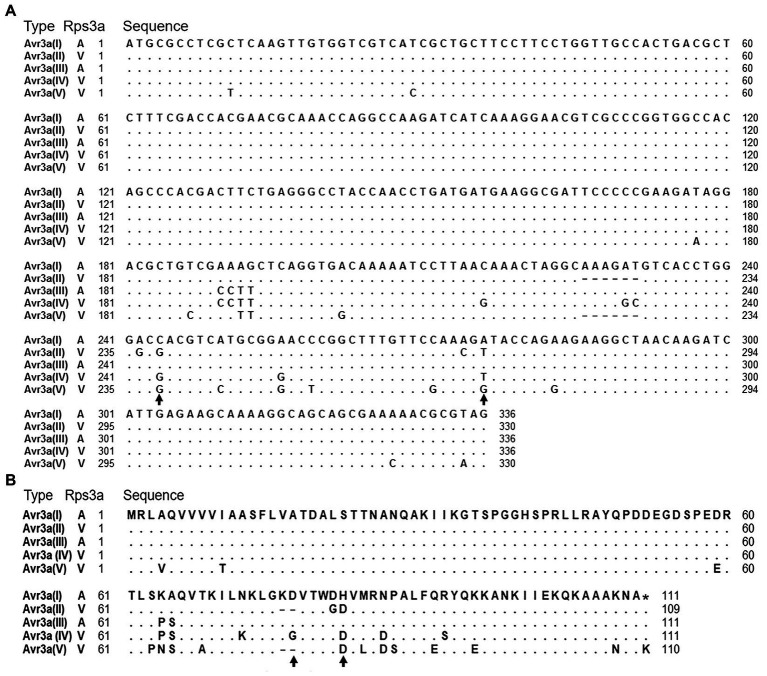
Sequence comparison of five *Avr3a* alleles. **(A)** Comparison of nucleotide sequences. Identities are indicated by dots and substitutions by the substituted nucleotides. Black arrows denote two nucleotides, which were different between avirulent alleles and virulent alleles. **(B)** Comparison of amino acid sequences. Black arrows denote two amino acids, which were different between avirulent alleles and virulent alleles.

To uncover the mechanism that *Avr3a* escapes recognition by *Rps3a*, sequence polymorphism of *Avr3a* was firstly investigated. A pair of *Avr3a* gene-specific primers, AVR3A1F/AVR3A1R (Supplementary Table S1), was used to amplify the full-length of *Avr3a*. The ~700bp fragments were amplified from all 32 isolates, cloned into a pMD19 vector, and sequenced. Five *Avr3a* alleles (GenBank accession numbers: MZ856318 to MZ856322) were identified in all sequenced isolates (Figure 1A), and two of them, *Avr3a*(II) and *Avr3a*(IV), were previously unreported alleles. Twenty-four mutations, including one deletion of six nucleotides and 23 single nucleotide polymorphisms (SNPs), were observed in the *Avr3a* coding region (Figure 1A). Two SNPs were synonymous substitutions, 20 SNPs were nonsynonymous substitutions, and one SNP caused a stop codon loss (Figure 1B). Among them, five SNPs were newly discovered mutations. Three new SNPs were from *Avr3a*(II), while the other two were from *Avr3a*(IV).

Virulence tests showed that all the isolates containing *Avr3a*(I) or *Avr3a*(III) were avirulent to *Rps3a* and all the isolates containing *Avr3a*(II), *Avr3a*(IV), or *Avr3a*(V) were virulent to *Rps3a* (Table 1). In the coding regions of *Avr3a*(II), *Avr3a*(IV), and *Avr3a*(V), there were 5, 10, and 17 mutations, respectively, (Figure 1A). These mutations in the *Avr3a* coding region could cause the gain of virulence of *P. sojae* to *Rps3a*.

### Transcriptional Analysis of *Avr3a* in *Phytophthora sojae* Isolates From China

**Figure 2:**
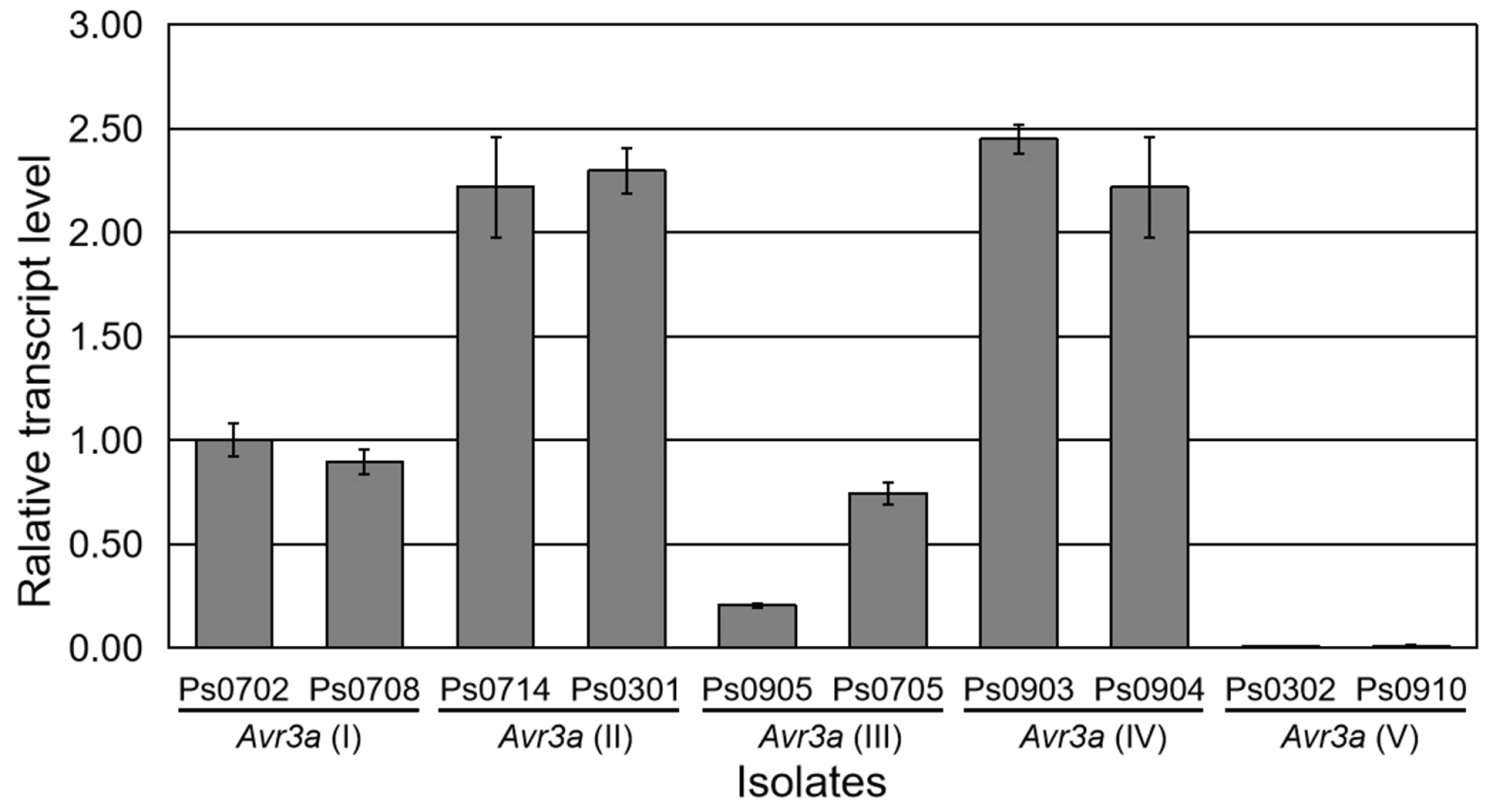
Transcriptional analysis of five *Avr3a* alleles. Expression levels relative to actin were calculated at 12h post-inoculation. The PCR reactions were replicated three times. Bars indicate standard errors.

The change in *Avr* gene expression is also an important strategy to escape recognization by *Rps* genes for *P. sojae*. To investigate whether the similar phenomenon for *Avr3a* occurs in this study, transcript levels of *Avr3a* were analyzed. A pair of gene-specific primers, AVR3A3F/AVR3A3R (Supplementary Table S1), was designed in the conserved regions of *Avr3a* to detect the transcript level of *Avr3a* using real-time RT-PCR. Ten isolates (Ps0702, Ps0708, Ps0714, Ps0301, Ps0905, Ps0705, Ps0903, Ps0904, Ps0302, and Ps0910), representative of five *Avr3a* alleles, were selected for transcriptional analysis. The results indicated that the *Avr3a* transcripts were detectable in the isolates containing *Avr3a*(I), *Avr3a*(II), *Avr3a*(III), and *Avr3a*(IV), but not in the isolates Ps0302 and Ps0910 containing *Avr3a*(V) (Figure 2). Transcriptional analysis suggested that the gain of virulence of the isolates Ps0302 and Ps0910 to *Rps3a* was due to the absence of *Avr3a*(V) transcript, not to DNA mutations in the coding regions.

### Sequence Polymorphisms in the Promoter and 5'-Untranslated Regions of *Avr3a*

Considering the absence of *Avr3a*(V) transcript, sequence polymorphisms in the promoter and 5′-UTR regions of *Avr3a* were further explored. Eight representative isolates (Ps0702, Ps0708, Ps0714, Ps0301, Ps0905, Ps0705, Ps0903, and Ps0904) and all 15 isolates containing *Avr3a*(V) were selected for polymorphism analysis. A pair of gene-specific primers, AVR3A2F/AVR3A2R, was used to amplify the promoter and 5′-UTR regions (1,000bp upstream from the start codon) of *Avr3a* (Supplementary Table S1). The ~1,100bp fragments were amplified from all selected isolates and then sequenced. No mutations were found in the 5′-UTR region of *Avr3a*. Eleven mutations, including two deletions, four insertions, and five SNPs, were observed in the promoter region of *Avr3a* (Table 2). Ten mutations were observed in the promoter region of *Avr3a*(V) whose transcripts were not detectable, whereas only two mutations were observed in the promoter regions of *Avr3a*(I), *Avr3a*(II), *Avr3a*(III), and *Avr3a*(IV) whose transcripts were detectable. Eight mutations, including one deletion, three insertions, and four SNPs, were unique to *Avr3a*(V) and were found in all 15 isolates containing *Avr3a*(V) (Table 2), suggesting that these mutations in the promoter region could result in the loss of *Avr3a*(V) transcription and cause the gain of virulence to *Rps3a*.

**Table 2:** Sequence polymorphism in the promoter and 5’-UTR regions of *Avr3a*.

Type of *Avr3a*	Isolate		Polymorphic position^1^									
		−962	−789	−761	−708	−671	−518	−417	−403 to −391	−337	−252	−238
I	Ps0702, Ps0708	T	T	A	A	A	C	G	GATGACGCACCCG	A	G	T
II	Ps0301, Ps0714	T	T	AGTGGAACAACA	A	A	C	G	GATGACGCACCCG	A	G	T
III	Ps0705, Ps0905	T	T	AGTGGAACAACA	A	A	C	G	GATGACGCACCCG	A	G	A
IV	Ps0903, Ps0904	T	T	A	A	A	C	G	GATGACGCACCCG	A	G	T
V	Ps0404, Ps0405, Ps0406, Ps0709, Ps0719, Ps0908, Ps0909, Ps0910, Ps0911	C	A	AGTGGAACAACA	C	AGCCTTCGCGAAGC CCTACTGTAGTGCA	T	GT	GATGACGCACCCG	AAGTAGGGCAG	–	T
	Ps0302, Ps0303, Ps0401, Ps0701, Ps0716, Ps0720,	C	A	AGTGGAACAACA	C	AGCCTTCGCGAAGC CCTACTGTAGTGCA	T	GT	–	AAGTAGGGCAG	–	T

1 *Polymorphic positions refer to the positions relative to the start codon. The transcription start site (TSS) is at−57*.

### Two SNP Markers for Virulent Alleles of *Avr3a*

*Avr3a*(I), *Avr3a*(II), *Avr3a*(III), and *Avr3a*(IV) were normally transcribed in *P. sojae* isolates. All the isolates containing *Avr3a*(I) or *Avr3a*(III) were avirulent to *Rps3a* and all the isolates containing *Avr3a*(II) and *Avr3a*(IV) were virulent (Table 1). Hence, *Avr3a*(I) and *Avr3a*(III) were avirulent alleles, while *Avr3a*(II) and *Avr3a*(IV) were virulent alleles. Transcribed virulent alleles of *Avr3a* were discovered for the first time. DNA mutations in the coding regions of *Avr3a*(II) and *Avr3a*(IV) caused the gain of virulence to *Rps3a*, which is a novel mechanism of virulence variation for *Avr3a*.

Although *Avr3a*(V) was not transcribed in *P. sojae* isolates, the previous study confirmed that *Avr3a*^P7064^, which is identical to *Avr3a*(V), did not interact with *Rps3a* by transient expression system, indicating that *Avr3a*(V) is also a virulent allele. Based on the evolutionary relationships of five *Avr3a* alleles (Supplementary Figure S1), these alleles were not able to be divided into avirulent and virulent groups. However, two SNPs, the 244th and 276th nucleotides, were identified which were different between two avirulent alleles and three virulent alleles (Figure 1A). The 244th nucleotide was a biallelic site, while the 276th nucleotide was a triallelic site. The 244th nucleotide was C in avirulent isolates and G in virulent isolates. Meanwhile, the 276th nucleotide was A in avirulent isolates and T/G in virulent isolates. These two SNP markers were able to differentiate virulent alleles from avirulent alleles accurately.

### An Improved PCR Assay for Detecting the Virulence of *Phytophthora sojae* to *Rps3a*

**Figure 3:**
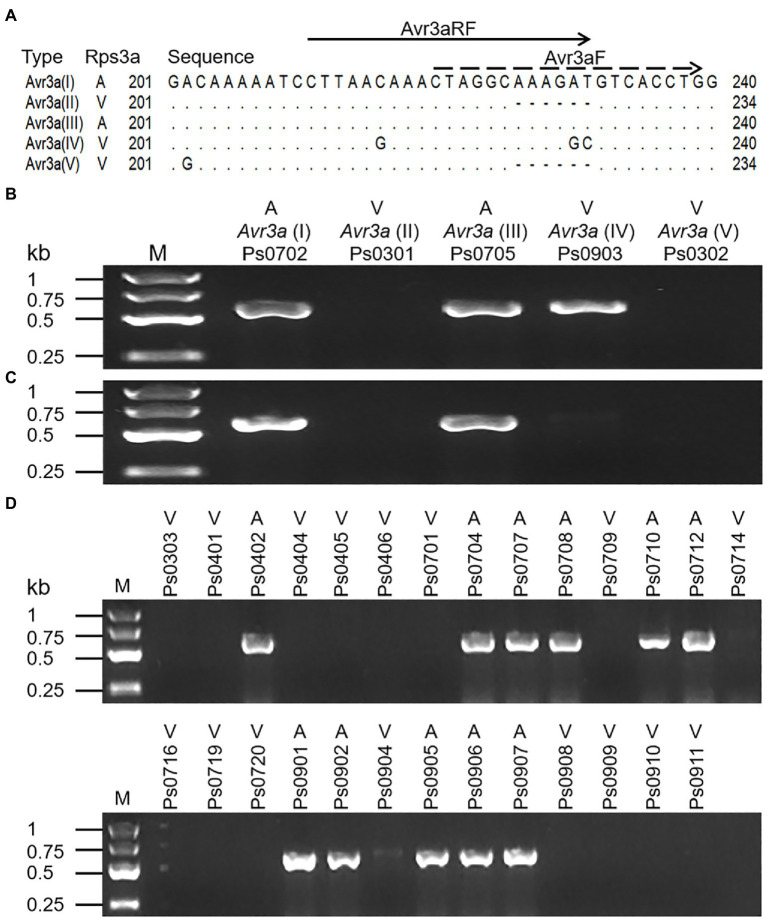
The PCR assays for the avirulence alleles of *Avr3a*. **(A)** Positions of the allele-specific forward primers. The dotted line shows the position of the allele-specific forward primer (Avr3aF) which designed by Dussault-Benoit et al. (2020). The solid line shows the position of the redesigned forward primer (Avr3aRF). The shared reverse primer (Avr3aR) is a gene-specific prime. A indicates an avirulent isolate or allele and V indicates a virulent isolate or allele. **(B)** The PCR assay of five *Avr3a* alleles with Avr3aF/Avr3aR. **(C)** The PCR assay of five *Avr3a* alleles with Avr3aRF/Avr3aR. **(D)** The PCR assay of 27 *P. sojae* isolates with Avr3aRF/Avr3aR.

Previously, a pair of allele-specific primers (Avr3aF/Avr3aR) designed by Dussault-Benoit et al. (2020) was used to detect the virulence of *P. sojae* to *Rps3a* by amplifying avirulent alleles of *Avr3a* (Supplementary Table S1). To determine if the primers are feasible for our isolates, five representative isolates (Ps0702, Ps0301, Ps0705, Ps0903, and Ps0302) in this study were tested. The ~600bp fragments were amplified from avirulent isolates Ps0702 and Ps0705. Regretfully, the same fragments were also amplified from virulent isolate Ps0903 with *Avr3a*(IV) (Figure 3B), despite using an annealing temperature of 62°C, suggesting that two SNPs of *Avr3a*(IV) in the middle position of the allele-specific forward primer (Avr3aF) could not prevent PCR amplification (Figure 3A). Given this, we designed a new allele-specific forward primer (Avr3aRF) to locate the two SNPs at the 3′ extremity (Figure 3A). The results from the improved PCR assay showed that the amplicons of *Avr3a* were present in two avirulent isolates but not in three virulent isolates (Figure 3C). The remaining 27 isolates were further tested to validate the accuracy of the improved PCR assay. The PCR results were completely consistent with the virulence of 27 isolates to *Rps3a* (Figure 3D), indicating that the improved PCR assay was accurate in detecting the virulence of all 32 isolates to *Rps3a*.

## Discussion

In this study, the polymorphism of *Avr3a* was analyzed detailedly in Chinese *P. sojae* isolates and a total of five *Avr3a* alleles were identified. Qutob et al. (2009) identified three *Avr3a* alleles in isolates P6497, ACR12 and fP7064. *Avr3a*^P6497^, *Avr3a*^ACR12^, and *Avr3a*^P7064^ are the same as *Avr3a*(I), *Avr3a*(III), and *Avr3a*(V) identified in the present study, respectively. Arsenault-Labrecque et al. (2018) identified two *Avr3a* alleles from 31 Canadian isolates. The two *Avr3a* alleles are the same as *Avr3a*(I) and *Avr3a*(V), respectively. *Avr3a*(II) and *Avr3a*(IV) are novel alleles that have not been described previously. In the coding region of *Avr3a*, a total of 23 SNPs were observed, including 20 nonsynonymous SNPs and two synonymous SNPs. The ratio of the nonsynonymous substitution to synonymous substitution (dN/dS) rate was far greater than one, suggesting that the *Avr3a* gene was subject to strong positive selection pressure in China. The result is in line with the high virulence frequency to *Rps3a* in *P. sojae* populations from China (Zhang et al., 2008; Cui et al., 2010; Tian et al., 2016; Wu et al., 2017). Twenty-eight pathotypes were identified in the 32 isolates, indicating that there was abundant virulence diversity among these isolates (Supplementary Table S2). It might be because all isolates were isolated from soil samples. Another 30 isolates which were isolated recently in China were used to clone and analyze the avirulence gene *Avr3a*, but no novel alleles were discovered.

Two avirulent and three virulent alleles of *Avr3a* were identified in this study. By comparing the amino acid sequences between avirulent and virulent alleles, the 77th and 82nd amino acids were identified as key amino acids (Figure 1B). The 77th amino acid was aspartic acid in avirulent alleles and glutamic acid/deletion in virulent alleles. The 82nd amino acid was histidine acid in avirulent alleles and aspartic acid in virulent alleles. The two amino acids may be very important for *Avr3a* in the interaction between *Avr3a* and *Rps3a*. Based on the analysis, we will study the influence of key amino acid variations of *Avr3a* on the recognition of *Rps3a* in the future.

Transcriptional analysis indicated that the *Avr3a* transcript was non-detectable in the isolates containing *Avr3a*(V). Further research showed that there were 10 mutations in the promoter region of *Avr3a*(V). Of them, eight mutations, including one deletion, three insertions, and four SNPs, were unique to *Avr3a*(V) compared with other alleles and were found in all isolates containing *Avr3a*(V). These mutations, especially the 10bp and the 27bp insertions, could be located in the transcription factor binding sites or transcription elements and result in the loss of *Avr3a*(V) transcription. Dong et al. (2011b) also analyzed the polymorphisms in the promoter region of *Avr3a*, but only one deletion and two insertions were observed between P6497and P7064. More polymorphisms were observed in Chinese *P. sojae* isolates. Of the 19 virulent isolates, 15 isolates belonged to this type, which was the main escape mechanism of *Avr3a* in China. *Avr3a*(II) and *Avr3a*(IV) were normally transcribed in *P. sojae* isolates, but the isolates with *Avr3a*(II) and *Avr3a*(IV) were virulent to *Rps3a*. Polymorphism analysis indicated that there were five mutations in the coding region of *Avr3a*(II), and there were 10 mutations in the coding region of *Avr3a*(IV). Therefore, *Avr3a*(II) and *Avr3a*(IV) should escape recognition by *Rps3a* through DNA mutations in the *Avr3a* coding region, which is a novel escape mechanism of *Avr3a*. Only four virulent isolates belonged to this type which was the secondary escape mechanism of *Avr3a* in China. Furthermore, Qutob et al. (2013) found that isolate ACR10 escaped recognition by *Rps3a* through small RNA-mediated gene silencing. A total of three different mechanisms, including DNA mutations in the coding region, loss of transcription, and small RNA-mediated gene silencing, were identified up to now. This research provides new insights into escape mechanisms of the *Avr3a* gene.

Virulence structure of *P. sojae* should be constantly monitored to determine and deploy effective *Rps* genes (Dorrance et al., 2016; Stewart et al., 2016). Conventional virulence tests are labor-intensive and time-consuming, thus a rapid and simple PCR assay for pathotype identification of *P. sojae* was developed (Dussault-Benoit et al., 2020). However, when the PCR assay was used to detect the virulence of isolate Ps0903 with *Avr3a*(IV) to *Rps3a* in this study, a false positive result occurred. The existence of *Avr3a*(IV) could also be one of the main causes that lead to five cases of discrepancy between the phenotyping assays and the PCR assays in the previous research. The improved PCR assay was developed by redesigning the allele-specific forward primer and eliminated the false positive. Even so, false positive results can still be obtained using the improved PCR assay, because some isolates with an avirulent allele could still be virulent to *Rps3a*. For instance, isolate ACR10 with *Avr3a*(I) was virulent to *Rps3a* due to small RNA-mediated gene silencing (Qutob et al., 2013). Therefore, we suggest that the *Avr3a* transcript levels of *P. sojae* isolates should be analyzed firstly by real-time RT-PCR and the isolates lacking *Avr3a* mRNA are directly identified as virulent isolates. Subsequently, the isolates with *Avr3a* mRNA were selected for virulence identification by using the improved PCR assay. The combination of the two assays could be the best strategy to accurately detect the virulence of all known isolates to *Rps3a*.

## Conclusion

In this study, two mechanisms of virulence variation for *Avr3a* were found in Chinese *P. sojae* populations. One was DNA mutations in the coding region of *Avr3a* that was a novel mechanism for *Avr3a*, and the other was the loss of transcription that was due to DNA mutations in the promoter region of *Avr3a*. In addition, three molecular markers were developed successfully for detecting the virulence of *P. sojae* to *Rps3a*. These findings may help us to understand how *P. sojae* overcome *Rps3a* at the molecular level in China and establish reliable molecular methods for pathotype identification of *P. sojae*.

## Data Availability Statement

The datasets presented in this study can be found in online repositories. The names of the repository/repositories and accession number(s) can be found at: https://www.ncbi.nlm.nih.gov/, MZ856318 to MZ856322.

## Author Contributions

LC designed the study, analyzed the data, and drafted the manuscript. YH and ZH performed all the experiments. YK reviewed and edited the manuscript. All authors have read and agreed to the published version of the manuscript.

## Funding

This work was supported by the grant U1804104 and U1404318 of the National Science Foundation of China.

## Conflict of Interest

The authors declare that the research was conducted in the absence of any commercial or financial relationships that could be construed as a potential conflict of interest.

## Publisher’s Note

All claims expressed in this article are solely those of the authors and do not necessarily represent those of their affiliated organizations, or those of the publisher, the editors and the reviewers. Any product that may be evaluated in this article, or claim that may be made by its manufacturer, is not guaranteed or endorsed by the publisher.
