# Supplementary material for: Mutations in the Promoter and Coding Regions of Avr3a Cause Gain of Virulence of Phytophthora sojae to Rps3a in Soybean
Source: Front Microbiol. 2021 Nov 11;12:759196. doi: 10.3389/fmicb.2021.759196 (PMC8632523; doi:10.3389/fmicb.2021.759196)
Supplement: Supplementary file 1 [file Table_1.DOCX]

**Supplementary** **Table S1.** The primers used in this study.

| Primer | Sequence (5' to 3') | Remark | Reference |
| --- | --- | --- | --- |
| AVR3A1F | ATCGAAGCCATATATCTACAGG | For amplifying and sequencing *Avr3a* |  |
| AVR3A1R | CCTACTGTATGCAAAGTGATCG |  |  |
| AVR3A2F | TGGCCAGCAATTACGAATTATAG | For amplifying and sequencing the promoter and 5'-UTR regions of *Avr3a* |  |
| AVR3A2R | GTGGCAACCAGGAAGGAAGC |  |  |
| AVR3A3F | TCTGAGGGCCTACCAACCTG | For real-time RT-PCR |  |
| AVR3A3R | GCTGCTGCCTTTTGCTTCTC |  |  |
| ACTAF | ACTGCACCTTCCAGACCATC | as a reference gene | Cui et al., 2012 |
| ACTAR | CCACCACCTTGATCTTCATG |  |  |
| Avr3aF | CTAGGCAAAGATGTCACCTG | For amplifying the avirulence alleles of *Avr3a* | Dussault-Benoit et al., 2020 |
| Avr3aR | ATCATGGCAAGCACCAATCT |  |  |
| Avr3aRF | CTTAACAAACTAGGCAAAGAT |  |  |
