# Supplementary material for: Mutations in the Promoter and Coding Regions of Avr3a Cause Gain of Virulence of Phytophthora sojae to Rps3a in Soybean
Source: Front Microbiol. 2021 Nov 11;12:759196. doi: 10.3389/fmicb.2021.759196 (PMC8632523; doi:10.3389/fmicb.2021.759196)
Supplement: Supplementary file 2 [file Table_2.DOC]

**Table S2.** Pathotypes of 32 *Phytophthora sojae* isolates.

| Isolate | Pathotype^1^ |
| --- | --- |
| Ps0402 | 2 |
| Ps0702 | 2,3b,4,6 |
| Ps0704 | 2,4 |
| Ps0707 | 1k,2,3b,3c,4,5,6 |
| Ps0708 | 3c,4,5,6,7 |
| Ps0712 | 1a,1b,1c,1k,2, |
| Ps0301 | 2,3a,3b,3c,4,5,6,7 |
| Ps0714 | 1a,1b,2,3a,3c,4,5,6,7 |
| Ps0705 | 2,3c,4,5,6 |
| Ps0710 | 1b,4,5,7 |
| Ps0901 | 2,4,5,6,7 |
| Ps0902 | 3c,5,6 |
| Ps0905 | 3b,3c |
| Ps0906 | 5 |
| Ps0907 | 3b,5 |
| Ps0903 | 2,3a,3c,4,5,6,7 |
| Ps0904 | 3a,3c,4,5,6,7 |
| Ps0302 | 1b,1d,2,3a,6 |
| Ps0303 | 1b,1d,2,3a,3b,3c,4,5,6,7 |
| Ps0401 | 2,3a,3b,3c,4,5,6 |
| Ps0404 | 1b,1d,2,3a,3b,4,5,6,7 |
| Ps0405 | 1b,1d,3a,3b,3c,4,5,6,7 |
| Ps0406 | 1a,1b,1c,1d,1k,3a,3b,3c,4,7 |
| Ps0701 | 3a,3c,4,5,7 |
| Ps0709 | 1b,2,3a,3c,4,5,6,7 |
| Ps0716 | 1b,1d,3a,3b,3c,4,5,6,7 |
| Ps0719 | 1b,2,3a,3b,3c,4,5,6,7 |
| Ps0720 | 1b,1d,2,3a,3b,3c,7 |
| Ps0908 | 1b,1d,2,3a,3b,3c,4,5,7 |
| Ps0909 | 1b,1d,2,3a,3b,3c,4,5,6,7 |
| Ps0910 | 1b,1d,2,3a,3b,3c,4,5,6,7 |
| Ps0911 | 1b,2,3a,3b,3c,4,5,6,7 |

^1^Pathotypes were identified using the hypocotyl split inoculation on soybean differentials Williams (universal susceptible), Harlon (*Rps*1a), L77-1863 (*Rps*1b), Williams79 (*Rps*1c), PI103091(*Rps*1d), Williams82 (*Rps*1k), L76-1988 (*Rps*2), L83-570 (*Rps*3a), PRX146-36 (*Rps*3b), PRX145-48 (*Rps*3c), L85-2352 (*Rps*4), L85-3059 (*Rps*5), Harosoy62xx (*Rps*6), and Harosoy (*Rps*7).
