# Supplementary figures and images for: Mutations in the Promoter and Coding Regions of Avr3a Cause Gain of Virulence of Phytophthora sojae to Rps3a in Soybean
Source: Front Microbiol. 2021 Nov 11;12:759196. doi: 10.3389/fmicb.2021.759196 (PMC8632523; doi:10.3389/fmicb.2021.759196)

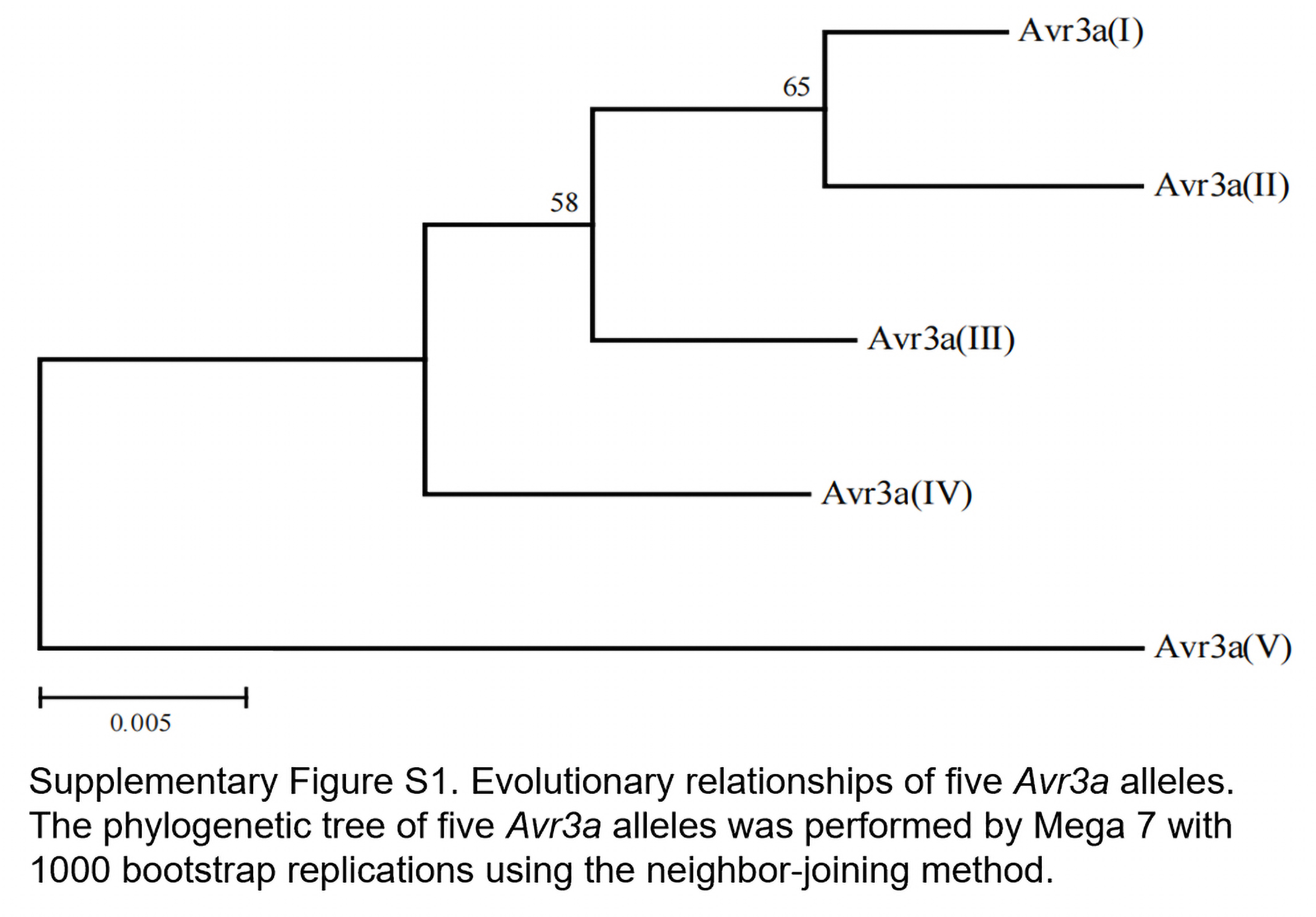

Supplement: Supplementary file 3 [file Image_1.JPEG]
